# Automatic Detection of the Pharyngeal Phase in Raw Videos for the Videofluoroscopic Swallowing Study Using Efficient Data Collection and 3D Convolutional Networks †

**DOI:** 10.3390/s19183873

**Published:** 2019-09-07

**Authors:** Jong Taek Lee, Eunhee Park, Tae-Du Jung

**Affiliations:** 1 Artificial Intelligence Application Research Section, Electronics and Telecommunications Research Institute (ETRI), Daegu 42994, Korea; jongtaeklee@etri.re.kr; 2 Department of Rehabilitation Medicine, Kyungpook National University Chilgok Hospital, Daegu 41404, Korea; teeed0522@hanmail.net; 3 Department of Rehabilitation Medicine, School of Medicine, Kyungpook National University, Daegu 41944, Korea

**Keywords:** action detection, action classification, 3D convolutional networks, pharyngeal phase, videofluoroscopic swallowing study

## Abstract

Videofluoroscopic swallowing study (VFSS) is a standard diagnostic tool for dysphagia. To detect the presence of aspiration during a swallow, a manual search is commonly used to mark the time intervals of the pharyngeal phase on the corresponding VFSS image. In this study, we present a novel approach that uses 3D convolutional networks to detect the pharyngeal phase in raw VFSS videos without manual annotations. For efficient collection of training data, we propose a cascade framework which no longer requires time intervals of the swallowing process nor the manual marking of anatomical positions for detection. For video classification, we applied the inflated 3D convolutional network (I3D), one of the state-of-the-art network for action classification, as a baseline architecture. We also present a modified 3D convolutional network architecture that is derived from the baseline I3D architecture. The classification and detection performance of these two architectures were evaluated for comparison. The experimental results show that the proposed model outperformed the baseline I3D model in the condition where both models are trained with random weights. We conclude that the proposed method greatly reduces the examination time of the VFSS images with a low miss rate.

## 1. Introduction

Dysphagia is a common clinical symptom that occurs between the mouth and the stomach where the patient suffers swallowing difficulty [1]. The prevalence of dysphagia is 30–50% in the elderly (≥65 years old), 40–80% in patients with stroke, 80% in patients with Alzheimer disease, 60% in patients with Parkinson disease, and 50% in patients with head and neck cancers [1,2,3]. Dysphagia is known to cause severe complications including malnutrition, dehydration, and aspiration pneumonia; these complications can lead to morbidity and mortality [2,3]. Aspiration pneumonia occurs in 43–50% of people during their first year of residency at a nursing home, with a mortality rate of up to 45% among residents with dysphagia [1,4].

**Figure 1:**
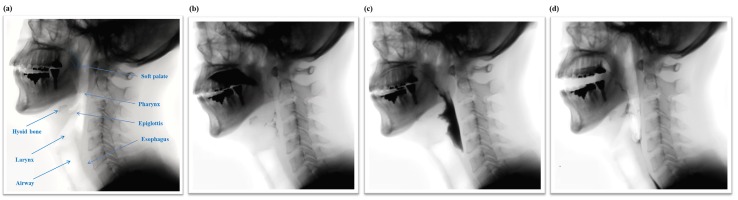
(**a**) Structural anatomy and normal physiologic swallowing of a thick liquid bolus in (**b**) the oral phase; (**c**) the pharyngeal phase; and (**d**) the esophageal phase.

The swallowing process is subdivided into three phases, the oral phase, the pharyngeal phase, and the esophageal phase, as shown in Figure 1. Swallowing is a rapid and complex function involving the coordinated contraction or inhibition of musculature in the mouth, tongue, larynx, pharynx, and esophagus [5,6]. In the oral phase, food is chewed and mixed with the saliva to form a bolus. The tongue pushes the bolus from anterior to posterior of the oral cavity via squeezing motion. Then, in the pharyngeal phase, the bolus is propelled from the oral cavity to the pharynx as the soft palate elevates and presses against the posterior wall of the pharynx. At this point, the hyoid bone and the larynx elevate and the epiglottis folds downward to protect the airway. This critical step makes the pharyngeal phase a crucial stage of swallowing as it prevents the transport of the bolus to the airway system. After the airway is protected, the tail of the bolus exits though opening of the upper esophageal sphincter. Finally, in the esophageal phase, the bolus passes down the esophagus to the stomach.

The videofluoroscopic swallowing study (VFSS) is the gold standard examination method to evaluate dysphagia [7]. When VFSS is administered, subjects are asked to swallow solid and liquid food mixed with radiopaque materials. Then through fluoroscopy, video data of the swallowing motion is obtained. Clinicians repeatedly examine the recorded video to assess any structural and functional abnormalities associated with swallowing and to confirm presence of airway protection during the swallowing process.

Although VFSS is the standard diagnostic tool of dysphagia, the evaluation of VFSS is a subjective interpretation based on visual inspection. In fact, a previous study reported that frame-by-frame analysis lacked intra-judge reliability for the assessment of biomechanical aspects of the pharyngeal phase [8]. Furthermore, clinicians could predict the risk of aspiration pneumonia during an inspection of abnormality in the pharyngeal phase in VFSS images [9]. However, the pharyngeal phase is a rapid sequence lasting one second or less in a normal swallowing process, and clinicians waste a lot of time selecting the time intervals of the pharyngeal phase [10,11].

To objectively assess VFSS image data, researchers have attempted to develop software tools for the clinicians who analyze VFSS [12,13,14]. To apply these tools, clinicians are required to provide specific time intervals of the pharyngeal phase and to annotate the region-of-interest (ROI) defined in a frame-by-frame analysis [10]. In this study, we aim to improve the labor-intensive procedures involved in analyzing VFSS images. We propose a novel system that automatically detects the pharyngeal phase of a swallowing process in raw VFSS video clips.

Our contribution in this paper is threefold. First, we propose a cascade framework that efficiently collects video clips of the pharyngeal phase for training the 3D convolutional networks. This framework no longer requires users to provide manual labels of the time intervals nor anatomical positions in the swallowing video. Second, we introduce an adaptation of Inflated Inception-V1 network architecture to improve classification and detection performance in cases in which pre-trained weights are not provided. Third, we propose a detection algorithm that integrates classification results from the trained 3D convolutional network, and provide evaluation of both classification and detection performances.

## 2. Related Work

To make an accurate biomechanical analysis of the swallowing process via VFSS images, some research groups have extracted the trajectory of the hyoid bone during the swallowing process. The first study proposed a biomechanical analysis of oral and pharyngeal structures during swallowing using VFSS images with the following steps: (1) digitizing each frame in a recorded video; (2) identifying a reference position; (3) drawing the anatomical points of interest in each image; (4) calculating the relative positions of the target against a reference position; and (5) plotting the movement of the target point through time [15,16]. However, manual tracking of an anatomical position is expensive in terms of time and human expertise.

Kellen et al. [12] designed a software program which could provide a 2D moving trajectory of the hyoid bone or larynx during swallowing in recorded images. A user defines an ROI that approximately overlays the hyoid bone which is known as the template. The template is tracked frame-to-frame throughout the image sequence. Similarly, Molfenter et al. [17] tried to analyze quantitative physiological variables related to the swallowing process using an image processing tool that is available for free. However, in clinical practice, these semi-automatic methods are still too costly and fully automatic algorithms are preferred.

Aung et al. [13] introduced a computer aided diagnosis system with minimal user input that can automatically determine anatomical positions based on several landmarks of the cervical spine vertebrae using the active shape model. After initializing the landmarks by user interaction, the registration process is applied to update the coordinates of the landmarks in each frame in order to compensate for the subject’s movement during the swallowing process. When the bolus passes by the landmarks such as hyoid bone [13] and epiglottis [11], a spatio-temporal plot can be generated. However, these methods are needed to demarcate the anatomical boundaries with user input.

Leonard [14] quantified pharyngeal residue on VFSS using the software platform Swallowtail (Belldev Medical, LLC, Arlington Heights, IL, USA). The algorithms used in Swallowtail are based on watershed segmentation that uses contours in the image intensity gradient to help define regions of related pixels. While successfully extracting mechanical measures for VFSS [18], this software platform is not able to autonomously extract the time intervals of interest.

Other studies applied a combination of VFSS with non-invasive sensors such as a microphone [19,20], the combination of a electromyography (EMG) with inertial measurement units (IMU) [21,22], and a piezoelectric sensor [23] to detect swallowing. Golabbakhsh et al. [19] reported that a non-invasive acoustic recording technique from a microphone located over the laryngopharynx could detect spontaneous swallowing. When compared to VFSS, the accuracy of this technique at detecting swallowing was 82%. Dudik et al. [20] proposed the use of cervical auscultation from a microphone to serve as a classification of swallowing using a multi-layer deep belief network. Imtiaz et al. [21] presented a wearable sensor system that was combined with both EMG and IMU for monitoring the movement of the head and neck axes during swallowing. Kalantarian et al. [23] introduced a wearable necklace which included an embedded piezoelectric sensor. To monitor eating habits for weight loss, this sensor can capture motion in the throat and transmit digital signals to a mobile application. These studies used machine-learning techniques to analyze digital signals from various sensors; however, these were not focused on the analysis of the physiologic swallowing process but on the detection of swallowing activities.

Recently, with the rapid progression of deep learning research on medical imaging, several deep learning based VFSS analysis methods have been suggested. Zhang et al. [10] developed a tracking system for hyoid bone detection using the single shot multibox detector, a state-of-the-art deep learning method for object detection. This method particularly focused on spatial region detection on a single image rather than on video data which consists of a sequence of images. Inspired by the recent success of 3D convolutional networks on action classification and action detection [24,25], researchers began to adopt these techniques to solve various problems such as understanding hyperspectral imagery [26,27], inferring the interaction forces between two objects from video [28], and VFSS analysis [29].

In a previous study [29], a system was able to classify whether a short VFSS video clip was in the pharyngeal phase or not. They used Inflated Inception-V1 from [25] as a pre-trained action classification architecture; however, this architecture was developed for classifying general human actions [30]. In this paper, we extend the framework for pharyngeal phase detection in raw VFSS video clips without the need for any manual annotations by modifying a general video classification architecture to capture rapid and small motions in the pharyngeal phase. We experiment with short clips in the order of thousands for classification and long clips in the order of hundreds for detection to show the robustness of the current framework. The comparison of the results of the current system with those of the previous version of our system are presented in the results section.

## 3. Dataset and Methods

**Figure 2:**
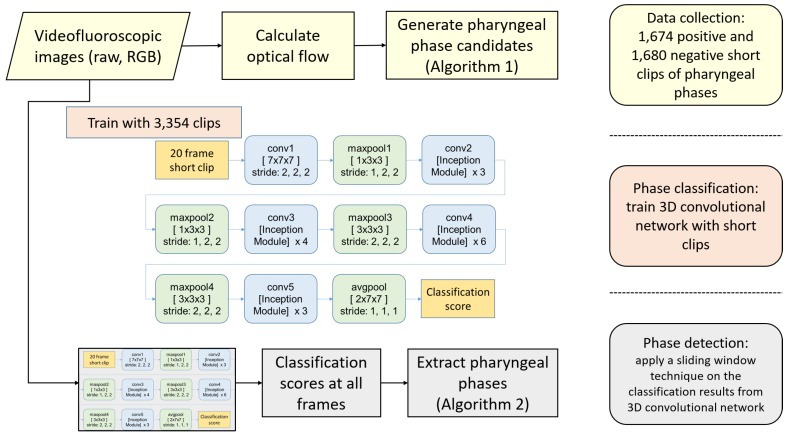
An overview of our framework.

We propose a three-stage framework to detect multiple occurrences of the pharyngeal phase in a long sequence of VFSS video. Figure 2 shows an overview of our framework, consisting of training clip generation, the video classification network using 3D convolutional layers, and the detection in raw video. In the first stage, we search all sequences of video frames to find short clips showing significant motion in the vertical direction. Because the food bolus flow during the pharyngeal phase is mostly visible in the motion map, the first stage is able to generate pharyngeal phase candidates with a small ratio of false negatives. As a result, we can efficiently collect most occurrences of the pharyngeal phase (92.0%). However, this stage suffers from a high quantity of false positives (50.1%) due to subjects’ other actions, such as coughing and movements involved in preparation to swallow, being mistaken for the pharyngeal phase. Therefore, in the second stage, we train 3D convolutional networks to classify such short clips, labelling whether each clip is in the pharyngeal phase or not. The I3D network [25] as baseline and its modified models are trained to compare the performance of different network architectures. In the third stage, we integrate the classification results on segmented clips using a sliding window technique to detect the pharyngeal phase in temporally untrimmed VFSS videos.

### 3.1. Dataset

The VFSS dataset was collected from 144 subjects who complained of subjective difficulties whilst swallowing and visited the inpatient and outpatient clinic of the Department of Rehabilitation Medicine at Kyungpook National University Chilgok Hospital from March to December in 2017. Subjects were 20 to 87 years old (mean age 63.2 ± 16.3 years) and included 100 males and 44 females. Subjects suffered from various medical conditions such as stroke and dementia (N = 52, 36.1%), elderly (N = 36, 25.0%), neuromuscular disease (N = 31, 21.5%), and cancer (N = 25, 17.4%). This retrospective study was approved by the Institutional Review Board at the Kyungpook National University Chilgok Hospital (IRB No. KNUCH 2018-05-006).

The recorded VFSS dataset was created by a clinician who performed the VFSS procedure according to the standard manual guidelines [7]. During the VFSS procedure, each subject, seated upright laterally in front of a fluoroscopy, swallowed one of the following eight substances which were mixed with diluted radio-opaque barium: 3, 6, and 9 mL of thin liquid (milk), thick liquid (fruit pudding), semi-solid (boiled rice), and solid (rice). Some subjects did not completely swallow all substances as they indicated severe aspiration or severe delayed swallowing reflex during the VFSS procedures. The camera recorded a lateral view of head and neck areas during the whole VFSS procedure.

**Figure 3:**
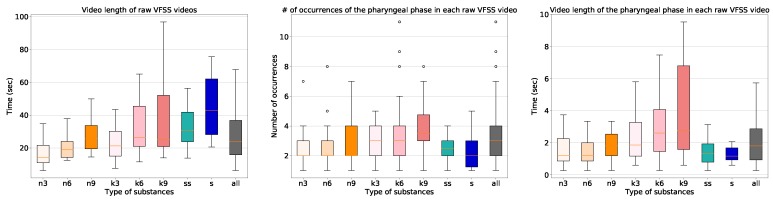
The box-plots of the video length of the raw videofluoroscopic swallowing study (VFSS) videos, the number of occurrences, and the video length of the pharyngeal phases in each raw VFSS video (from left to right). On the horizontal axes, n, k, ss, and s indicate thin liquid, thick liquid, semi-sold, and solid, respectively. The labels 3, 6, 9 indicate the amount of substance used in the experiment in milliliters.

The characteristics of the dataset are shown in Figure 3. The length of the raw video clips varied significantly from eight seconds to five minutes with a median value of 24 s. The frame rate of the videos that we collected is 30 frames per second (FPS), and we sampled frames at 15 FPS for all processing. The VFSS procedure using the thin liquid substance required the least time, and the VFSS procedure using the solid substance required the most time. In addition, the procedures involving the larger amounts of the substances took longer. The number of occurrences of the pharyngeal phase was fairly uniform. All of the collected 1085 long video sequences contain at least one swallowing event including the pharyngeal phase, with a variety of types of substances.

### 3.2. Efficient Training Data Collection by Generating Pharyngeal Phase Candidates Using Optical Flow

In order to train the video classification network, we first needed to collect training video data. Because of a memory issue related to 3D convolutional networks, it is not feasible to process a raw video of a large number of frames. Because the pharyngeal phase is usually very short (about one second), we decided to collect 20 frame short clips. As the pharyngeal phase is only a small part in a raw VFSS video, it is inefficient to randomly collect from a raw video. Therefore, we propose an algorithm for pharyngeal phase candidate generation to efficiently collect pharyngeal phase candidates using optical flow.

Algorithm 1 shows the pseudo-code of the candidate generation algorithm. First, we applied a TV-L1 optical flow algorithm [31,32] on gray-scale images. The optical flow values were truncated to the range −10 to 10, and divided by 10. If the maximum of the absolute values in the Y component in the center region of the frame was larger than the threshold ($f_{th}=0.4$ is applied in this paper), a vote was given on the frame and its eight nearest frames. After the voting process, the vote list was sorted in descending order. We scanned the vote list for all times *t* until collecting five candidates satisfying two conditions: the number of the vote at frame *t* is greater than the threshold ($v_{th}=2.5$ is applied in this paper), and the frame *t* is not included in other candidates. 

**Algorithm 1:** Pharyngeal phase candidate generation

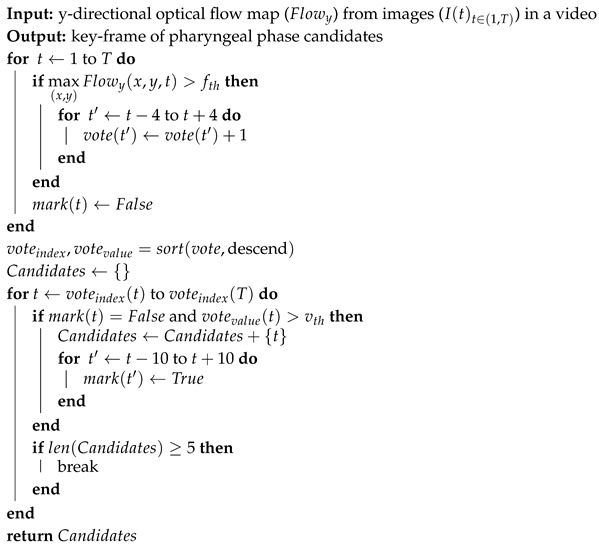



**Figure 4:**
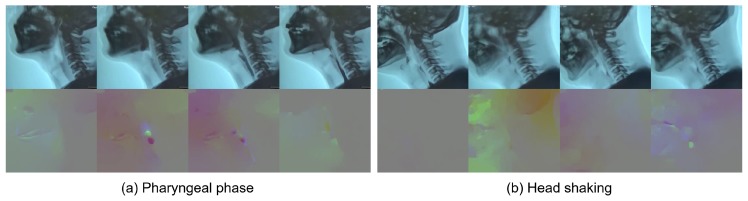
RGB (top) and optical flow (bottom) visualization of (**a**) positive sample—pharyngeal phase; and (**b**) negative sample—other action (e.g., head motions due to coughing).

After the selection process, 3354 short clips of pharyngeal phase candidates were generated from 1085 long video sequences. Each video sequence had at least one pharyngeal phase occurrence. However, no candidates for pharyngeal phase intervals were generated in 88 out of 1085 video sequences. A total of 3354 short clips were labeled as being the pharyngeal phase (1674 samples) and others (1680 samples). As shown in Figure 4, RGB and optical flow visualization of the pharyngeal phase candidates shows the complexity of the optical flow analysis of subjects’ movements. The data for the video classification was divided into two sets, 2696 for training and 658 for validation.

### 3.3. Training 3D Convolutional Networks Using Rgb/Optical Flow/Joint

Our baseline network was the Inflated Inception-V1 from the I3D networks [25]. The input video clip contains 20 frames, each frame being resized to 224 × 224. The number of channels was three for the RGB input stream and two for the optical flow input stream. While Lee and Park [29] trained their network with step learning rate decay, we used cosine learning rate decay with warm-start [33] to stabilize training. The initial learning rate was 0.1 and mini-batch size was 6. The models with and without pre-trained weights on the Kinetics dataset [30] were trained to compare the effect of pre-training.

While the Inflated Inception-V1 architecture achieved a state-of-the-art performance in action classifications such as UCF-101 [34], HMDB-51 [35], and Kinetics [30], we modified the architecture to improve the performance in classification on the VFSS videos as shown in Table 1. The proposed architecture is inspired by ResNet50 [36], which used 3, 4, 6, 3 residual blocks to build 50 layers. Instead of residual blocks, we used the inception module that is the concatenation of [1×1×1 conv], [1×1×1 conv, 3×3×3 conv], [1×1×1 conv, 3×3×3 conv], and [3×3×3 maxpool, 1×1×1 conv].

Unlike human behavior, the bolus flow in the VFSS videos is a small part of the entire image and is fast in motion. For this reason, we added more inception modules at the early stage of the video classification to watch such small changes more closely. As a result, the number of parameters and the training time of the proposed architecture increased by 15.8% and 19.0%, respectively. However, the classification and detection performance significantly improved, as shown in Section 4.2. The training performances of the pre-trained model and the model with random weights were saturated near 8 K and 20 K iterations, respectively.

### 3.4. Detection Algorithm for Raw Video Sequences Using Trained 3D Convolutional Networks

To detect the pharyngeal phase in the raw VFSS video sequences, we integrated the classification results from 3D convolutional networks in consecutive frames using a sliding window technique. The window and stride sizes were set to 20 and 5 frames, respectively. This setup allowed for a dense search of the pharyngeal phase with the moderate inference time of 3D convolutional classifiers. For all 215 test video clips (1 h 50 min duration), the inference time for the sliding window technique was only 21 min.

The details of the proposed detection algorithm are presented in Algorithm 2. Firstly, a smoothing filter is applied on the classification results to reduce noise and mis-classification. Then, all frames are scanned, a frame being marked if the score of the frame is higher than the threshold, $score_{th}$, as long as that frame has not yet been marked. When the score of one of the following frames is no longer higher than the threshold, $score_{th}$, and the number of the frame is larger than the threshold, $frame_{th}$, the start frame index, end frame index, and confidence score are calculated. Finally, the detection results are saved and searching continues until the end of a video. There are three hyper-parameters in Algorithm 2, $\left[score_{th},frame_{th},\lambda \right]$, and we found that $\left[score_{th}=0.5,frame_{th}=5,\lambda =0.001\right]$ functioned properly.

**Algorithm 2:** Sliding window technique-based pharyngeal phase detection

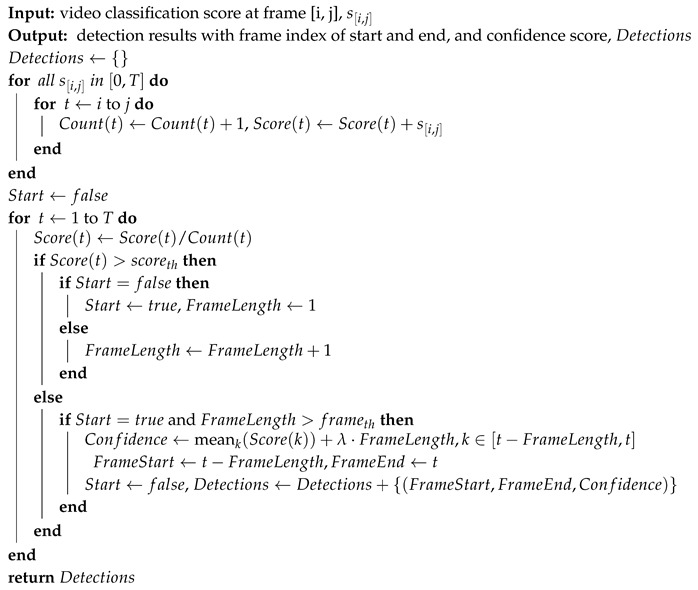



## 4. Experiments and Results

### 4.1. Evaluation Metrics and Ground Truth Generation

In order to evaluate the performance of the pharyngeal phase detection methods, we measured the detection F1-score and the detection time error. The performance measurement in terms of detection with the factors of time and space is more complex than that of object detection in an image. For object detection in an image, intersection over union (IOU) is measured to evaluate the overlap between two bounding boxes (one is from predicted detections and the other is from ground-truth detections). For phase detection in a video, IOU is similarly measured by evaluating the frame overlap between two time-predicates. True-positive (TP) is considered as a correct detection when the IOU of a predicted detection and the true detection are larger than the threshold (set to 0.3 in this experiment). The IOU of two time-predicates can be calculated by the number of overlapping frames between two predicates divided by the number of union frames between them.

Each predicted detection will only be a TP for one true detection with the lowest detection time error. Each true detection will only be a TP for one predicted detection with the highest confidence and IOU larger than the threshold. The predicated detection will be ignored if there are true detections with IOU > threshold but all the true detections are considered as TPs by other predicted detections with higher confidence. A false-positive (FP) is a predicted detection if there is no true detection with IOU > threshold. Finally, a false-negative (FN) is a true detection that was not predicted.

Precision is the proportion of TPs out of all predicted detections, and recall is the proportion of TPs out of all true detections as in Equations (1) and (2). The F1-score is the harmonic mean of the precision and recall, as shown in Equation (3). Finally, the detection time error is calculated as the average of the absolute time error of the start, middle, and end position of the detected phase occurrences from the ground truth.

(1) $$\begin{array}{cc} precision & =\frac{TP}{TP+FP}=\frac{TP}{\mathrm{predicted}\;\mathrm{detections}} \end{array}$$

(2) $$\begin{array}{cc} recall & =\frac{TP}{TP+FN}=\frac{TP}{\mathrm{true}\;\mathrm{detections}} \end{array}$$

(3) $$\begin{array}{cc} F_{1} & =\frac{2\cdot precision\cdot recall}{precision+recall} \end{array}$$

**Figure 5:**
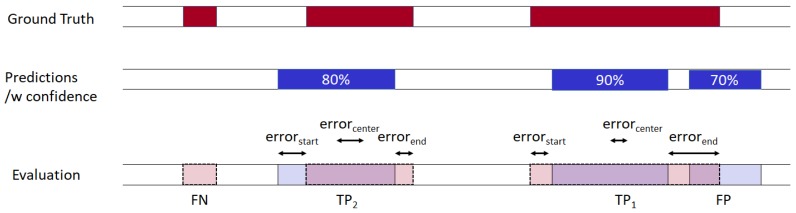
An evaluation of the sample detection results.

A sample evaluation process is shown in Figure 5. There are three occurrences of the pharyngeal phase in the ground truth, and our algorithm predicted three occurrences with confidence score. In the evaluation process, the detection with the highest confidence score searches the ground truth time predicate which has not yet been chosen by another detection and has the highest IOU value (>threshold). Any ground truth which is not predicted by the algorithm is counted as an FN, and a predicted detection which could not find an unmatched ground truth is counted as an FP. For all TPs, we measured errors at the start, middle, and end of the time predicates.

Two expert clinicians validated in the pharyngeal phase were employed; they annotated the class of the short VFSS clips (for classification) and the start and end frames of all the occurrences of the pharyngeal phase in the raw VFSS clips (for detection) without any prior knowledge. The beginning of the pharyngeal phase is defined as the point when the head of bolus is propelled to the pharynx, when the soft palate elevates and presses against the posterior wall of the pharynx [6,37]. The end of the pharyngeal phase is defined as the point when the tail of bolus exits through the opening of upper esophageal sphincter [6,37]. For classification, the ground truth label (pharyngeal phase or not) was finally determined by the employed clinicians reviewing their results and coming to an agreement if the annotation mismatched. For detection, the ground truth label (start frame and end frame) was simply determined by averaging the two results.

### 4.2. Results

We evaluated the performance of the baseline I3D network with pre-trained weights, baseline I3D network without pre-trained weights and the proposed network without pre-trained weights. In addition, we also compared the performance of the networks that use RGB against networks that use optical flow stream, as shown in Table 2. First, the training time with the minimum training error was measured. The networks using RGB required 50–100% more training time than the networks that use optical flow. Furthermore, the pre-trained models required about 40% of the training time of the models with random weights.

Although the training time for the optical flow network was shorter than the training time of the RGB network, the classification accuracy was higher in the RGB network than that of the optical flow network. While optical flow is very informative and effective at providing distinctive directions, it can also be inherently noisy due to it capturing subjects’ movement as well as external noise factors in the video recording process. Considering the computational cost of calculating optical flow, RGB stream would be the favorable choice for both accuracy and speed. As shown in Table 2, the detection error is typically much higher than the classification error. We also report that the detection error becomes significantly larger with a small increase in the error in classification.

The proposed architecture that uses RGB stream (bold in Table 2) showed the best performance among the networks that use random initial weights. This model performed with 73.21% in detection F-1 score, with 71.69% in precision and 74.80% in recall. The detection time error of the proposed model using RGB was 2.01 s, which is shorter than the error of the baseline networks. In addition, the proposed model outperformed the baseline model in both cases using RGB and optical flow.

The pre-trained model that used RGB stream (italics in Table 2) showed the best performance among all the tested models. Training from pre-trained weights rather than from random weights was advantageous for training time, accuracy in classification and accuracy in detection. However, because training models with large datasets such as the Kinetics dataset [30] require a high computational cost, the proposed model is most advantageous when resources are limited and only moderate performance is required.

**Figure 6:**
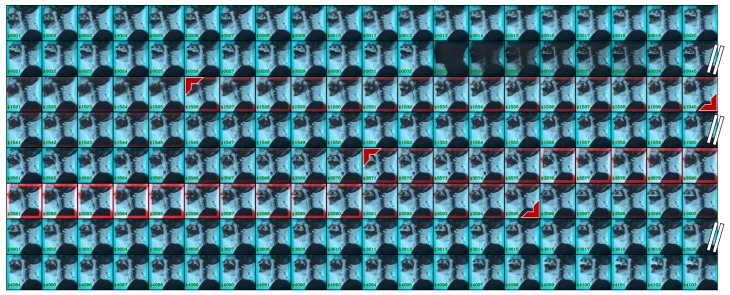
Detection results on a raw VFSS video. The detected occurrences of the pharyngeal phase are indicated by the red colored right angle bracket. From a total of 4103 frames, 160 frames are selectively displayed.

Figure 6 shows an example of the detection results by our system on raw VFSS videos. The frame number is shown in the left bottom corner of each image. A selection of sample frames are presented as the number of total frames is over 4000. The red boxes on the images are determined by the detection confidence score. In the presented four-minute video, our system found two occurrences of the pharyngeal phase. The two occurrences last for a total of three seconds, and both of them were correctly detected within a 0.27 s time error.

## 5. Discussion

We propose a novel framework that consists of three stages: (1) generation of pharyngeal phase candidates using optical flow; (2) training of the candidates using a 3D convolutional network; and (3) application of a sliding window technique to detect the pharyngeal phase during a swallow in VFSS images. This study aims to present a system that identifies the pharyngeal phase in VFSS video clips without the need for spatial or temporal annotations. This model was validated on a large clinical dataset.

Our framework with the proposed architecture predicted 466 true positive occurrences of the pharyngeal phase with 157 false negatives and 184 false positives from 215 raw VFSS videos. With the use of our proposed framework, the total video length for the VFSS analysis significantly reduced from 110 min to 10 min. To compensate for the time error in detection task, we extended the time window of the detected time window by 10 frames from both the start and end of the time predicates. Because detection time error results in this extended time cost for the VFSS analysis, our focus for the future work will be on the reduction of the detection time error. In 18 out of the 215 videos, the system missed all the occurrences of the pharyngeal phase in the raw videos. The miss rate was as low as 8.4%, however, this result increased the analysis time for the VFSS by eight minutes.

An aspiration event is defined as the instance where material passes below the vocal cord and enters the airway during the pharyngeal phase. Because the automatic aspiration detection is beyond the scope of this paper, we compared the performance of aspiration detection by expert clinicians with the pharyngeal phase detection results of our framework and ground truth. The number of detected aspiration events from 215 raw videos was 23 by expert clinicians and 20 by the proposed algorithm.

The pharyngeal phase is a rapid and complex motion. As food bolus movement from the oral cavity to the esophagus triggers the swallowing reflex or swallowing response, the coordinated physiological events occur in rapid overlapping sequence [5]. To prevent the food bolus from entering the airway, a coordinated movement of laryngeal elevation by suprahyoid muscles and closure of the larynx by epiglottic inversion occur in the pharyngeal phase [5]. Our framework automatically trained these complex swallowing movements without spatial annotations such as anatomical structures in the VFSS images.

Automatic detection of the pharyngeal phase could be useful for clinical examination of VFSS images. The pharyngeal phase is a critical stage of the swallowing process as abnormality in it can cause serious medical complications such as aspiration pneumonia or asphyxia [5]. To assess the pharyngeal phase in VFSS images, clinicians manually search for the pharyngeal phase in the VFSS images through visual inspection. Previously developed software applications and computer assisted analysis programs of VFSS images require manual annotations to select the time intervals of interest during the swallowing process [10,11,12,13,15,16,29]. These preparations related to the specific time intervals are costly. In contrast, our novel framework provides clinicians with clips of interest, specifically, the pharyngeal phase, taken from a complete VFSS image without the need for temporal annotations. Our framework is expected to reduce time expenses for VFSS analysis for clinicians who need to search for the presence of aspiration in the pharyngeal phase.

There are some limitations to this study. First, we did not detect the oral and esophageal phases in the swallowing process. Further studies are needed to modify our framework to detect the oral and esophageal phases in VFSS images. Second, our method does not capture pre-swallow or post-swallow aspiration, as the pharyngeal phase is the only phase of interest in this framework. Further investigations are needed to more effectively identify the presence of aspiration during the swallowing process.

## 6. Conclusions

This study presents a novel framework which can detect the pharyngeal phase during the complex process of swallowing without any manual adjustments. This framework could play a crucial role in terms of developing fully automatic applications for the analysis of VFSS images.

## Figures and Tables

**Table 1:** The Inflated Inception-V1 architectures and proposed architecture.

Layer Name	Inflated Inception-V1	Proposed
conv1	[7×7×7] stride: 2, 2, 2	[7×7×7] stride: 2, 2, 2
maxpool1	[1×3×3] stride: 1, 2, 2	[1×3×3] stride: 1, 2, 2
conv2	[1×1×1], [3×3×3]	[Inception Module] ×3
maxpool2	[1×3×3] stride: 1, 2, 2	[1×3×3] stride: 1, 2, 2
conv3	[Inception Module] ×2	[Inception Module] ×4
maxpool3	[3×3×3] stride: 2, 2, 2	[3×3×3] stride: 2, 2, 2
conv4	[Inception Module] ×5	[Inception Module] ×6
maxpool4	[3×3×3] stride: 2, 2, 2	[3×3×3] stride: 2, 2, 2
conv5	[Inception Module] ×2	[Inception Module] ×3
avgpool	[2×7×7] stride: 2, 2, 2	[2×7×7] stride: 1, 1, 1
# of parameters	1.228 M	1.422 M

**Table 2:** Accuracy rates of inflated inception-V1 with/without pre-trained weights and the proposed architecture using RGB and optical flow. The proposed architecture using the RGB stream (in bold) showed the best performance among all the networks using random weights, and the pre-trained model with the RGB stream (in italics) showed the best performance among all the tested models.

Method	Training Time	Classification Accuracy	Detection F-1 Score	Detection Time Error
Inception-V1/RGB/random	5.8 h	95.05%	63.05%	2.40 s
Inception-V1/Flow/random	3.1 h	92.21%	45.73%	3.82 s
**Proposed/RGB/random**	**6.9 h**	**95.98%**	**73.21%**	**2.01 s**
Proposed/Flow/random	3.5 h	93.19%	49.51%	3.43 s
*Inception-V1/RGB/pre-trained*	*2.1 h*	*96.74%*	*84.25%*	*1.42 s*
Inception-V1/Flow/pre-trained	1.3 h	96.20%	80.25%	1.99 s
